# Functionally distinct roles for T and Tbx6 during mouse development

**DOI:** 10.1242/bio.054692

**Published:** 2020-08-27

**Authors:** Amy K. Wehn, Deborah R. Farkas, Carly E. Sedlock, Dibya Subedi, Deborah L. Chapman

**Affiliations:** Department of Biological Sciences, University of Pittsburgh, Pittsburgh, PA 15260, USA

**Keywords:** T, Tbx6, Brachyury, Mouse, T-box, Mesoderm

## Abstract

The mouse T-box transcription factors T and Tbx6 are co-expressed in the primitive streak and have unique domains of expression; T is expressed in the notochord, while Tbx6 is expressed in the presomitic mesoderm. T-box factors are related through a shared DNA binding domain, the T-domain, and can therefore bind to similar DNA sequences at least *in vitro*. We investigated the functional similarities and differences of T and Tbx6 DNA binding and transcriptional activity *in vitro* and their interaction genetically *in vivo*. We show that at one target, *Dll1*, the T-domains of T and Tbx6 have different affinities for the binding sites present in the mesoderm enhancer. We further show using *in vitro* assays that T and Tbx6 differentially affect transcription with Tbx6 activating expression tenfold higher than T, that T and Tbx6 can compete at target gene enhancers, and that this competition requires a functional DNA binding domain. Next, we addressed whether T and Tbx6 can compete *in vivo*. First, we generated embryos that express Tbx6 at greater than wild-type levels embryos and show that these embryos have short tails, resembling the *T* heterozygous phenotype. Next, using the dominant-negative *TWis* allele, we show that *Tbx6+/− TWis/+* embryos share similarities with embryos homozygous for the *Tbx6* hypomorphic allele *rib-vertebrae*, specifically fusions of several ribs and malformation of some vertebrae. Finally, we tested whether Tbx6 can functionally replace T using a knockin approach, which resulted in severe *T* null-like phenotypes in chimeric embryos generated with ES cells heterozygous for a *Tbx6* knockin at the *T* locus. Altogether, our results of differences in affinity for DNA binding sites and transcriptional activity for T and Tbx6 provide a potential mechanism for the failure of Tbx6 to functionally replace T and possible competition phenotypes *in vivo*.

## Abstract (precis)

**Summary:** Mouse Tbx6 fails to compensate for heterozygous loss of T; instead ectopic Tbx6 in the *T* expression-domain in knockin embryos generates *T* null-like phenotypes suggestive of competition.

## INTRODUCTION

The T-box proteins constitute a family of transcription factors that are related through a shared DNA binding domain, the T-domain that allows family members to bind similar DNA sequences. Therefore, these related factors have the potential to regulate the expression of the same target genes. However, T-box factors may differ in how they regulate transcription once they bind to DNA; acting as transcriptional activators, repressors or as both. Interestingly, in addition to facilitating DNA binding, the T-domain can also interact with chromatin remodelers (Beisaw et al., 2018; Istaces et al., 2019; Lewis et al., 2007; Miller et al., 2008, 2010), including histone methyltransferases, demethylases, acetyltransferases and deacetyltransferases, and these interactions regulate the permissiveness of the chromatin environment. Outside of the T-domain, the proteins share little similarity. T-box transcription factors are indispensable for normal development of organisms ranging from worms to humans. Homozygous loss of these family members can have catastrophic effects on the developing embryos often leading to lethality with phenotypes highlighting the importance of these proteins in diverse processes, including cell proliferation, migration, cell fate and tissue morphogenesis (reviewed in Papaioannou, 2014). Interestingly, heterozygosity for T-box factors can also have phenotypic consequences. For example, the founding member of this family, Brachyury or T, was initially identified by the short-tailed heterozygous phenotype (Dobrovolskaia-Zavadskaia, 1927). In humans, these heterozygous conditions can lead to syndromes, including Holt-Oram Syndrome (HOS, *TBX5*), ulna mammary syndrome (UMS, *TBX3*), DiGeorge syndrome (*TBX1*), spondylocostal dysostosis (*TBX6*) and cleft palate and ankyloglossia (*TBX22*) (reviewed in Ghosh et al., 2017). Therefore, maintaining the proper levels of these transcription factors is also critical for normal development.

In the mouse, T and Tbx6 are critical for mesoderm formation and differentiation. *T* is expressed in the notochord and primitive streak (PS) with T expression downregulated as cells leave the streak (Wilkinson et al., 1990). Likewise, *Tbx6* is expressed in the PS but is also expressed in the presomitic paraxial mesoderm (PAM) with expression being downregulated as the somites are formed (Chapman et al., 1996). As previously stated, heterozygosity for *T* results in loss of posterior structures resulting in variable (shortened) tail lengths. Homozygous loss of *T* leads to more pronounced axis truncations, with the embryonic axis terminating just caudal to the forelimb; embryonic lethality by embryonic day (e) 10.5 is due to the failure to form the extraembryonic allantois (Herrmann et al., 1990). These variable phenotypes for the *T* hetero- and homozygous null embryos suggest that different levels of T are required along the axis, with highest T levels required for more posterior development (MacMurray and Shin, 1988; Stott et al., 1993). The dosage sensitivity of the axis to T levels is not limited to mice as bobtail dogs (Haworth et al., 2001) and Manx cats (Buckingham et al., 2013) also display short tails when heterozygous for *T* mutations. Development is also sensitive to Tbx6 levels; spondylocostal dysostosis in humans can be caused by mutations in TBX6 that reduce its transcriptional activity (Sparrow et al., 2013). We and others have further shown that the spontaneous mouse mutant *rib-vertebrae* is a *Tbx6* regulatory mutation that results in decreased levels of *Tbx6* expression, and fusions of the ribs and vertebrae and shortening of the axis due to vertebral malformations (Watabe-Rudolph et al., 2002; White et al., 2003). Homozygous loss of *Tbx6* results in the improper patterning of ∼9 anterior somites and the replacement of more posterior PAM with neural tissue (Chapman and Papaioannou, 1998). *Tbx6* is initially expressed in the *T* null, but expression is lost once the mutant phenotype becomes obvious (Chapman et al., 1996). *T* continues to be expressed in the enlarged tail bud region of the *Tbx6* mutant (Chapman and Papaioannou, 1998). This data suggests that neither T nor Tbx6 can compensate for the loss of the other in these mutant situations.

We sought to examine why these related factors could not compensate for each other despite sharing a similar DNA binding domain and both functioning as transcriptional activators. We hypothesized that there were differences in their binding and activity that contributed to this failure to compensate. We first tested the binding affinities of T and Tbx6 for T binding sites in a known target for both, *Dll1*. We then examined how each affects transcription from several enhancers, including enhancers of *in vivo* targets. These results suggest not only different binding affinities and transcriptional activity, but also that these related factors can compete with each other and that competition is dependent on the T-domain. Given this *in vitro* competition, we examined the effect of overexpressing Tbx6 in its endogenous domain and the *T/Tbx6* genetic interactions using the *T^Wis^* dominant allele and *Tbx6* loss-of-function allele. In both situations we found evidence that suggested competition, specifically overexpression of Tbx6 resulted in T-like short tail phenotypes, while *T^Wis^*/+ *Tbx6+/−* embryos share similarities with *Tbx6* hypomorphs. Finally, we tested the ability of Tbx6 to functionally replace T using a knockin approach in mice. We found that Tbx6 was not sufficient to rescue a heterozygous loss of T when Tbx6 is expressed in the T endogenous domain. Moreover, we found that *Tbx6* expression in the T domain inhibited normal development of chimeric embryos. Altogether, our results suggest that T and Tbx6 differentially regulate downstream target gene expression, through either DNA binding affinities, transcriptional activity or both, that they can compete at some targets, and that this competition is mediated by the DNA binding domain.

## RESULTS

### T and Tbx6 DBD have different affinities for T-box binding sites in the Dll1-msd enhancer

Genetic, biochemical and transcriptional assays demonstrated that T-box and Wnt signaling are critical for controlling *Dll1* expression in the PSM (Beckers et al., 2000b; Hofmann et al., 2004; White and Chapman, 2005; White et al., 2003). *Dll1* is a target of both T and Tbx6 (Hofmann et al., 2004; White and Chapman, 2005). Beckers and colleagues identified a *Dll1 ‘msd’* enhancer element capable of driving *lacZ* reporter expression in the mouse PSM (Beckers et al., 2000a). This *Dll1-*msd enhancer contains T-box and TCF/LEF binding sites that are required for enhancer activity *in vitro* and *in vivo* (Hofmann et al., 2004; White and Chapman, 2005). To further understand similarities and differences between T and Tbx6 target gene regulation, we first measured the affinities of the T and Tbx6 T-domain for these binding sites.

**Fig. 1.**
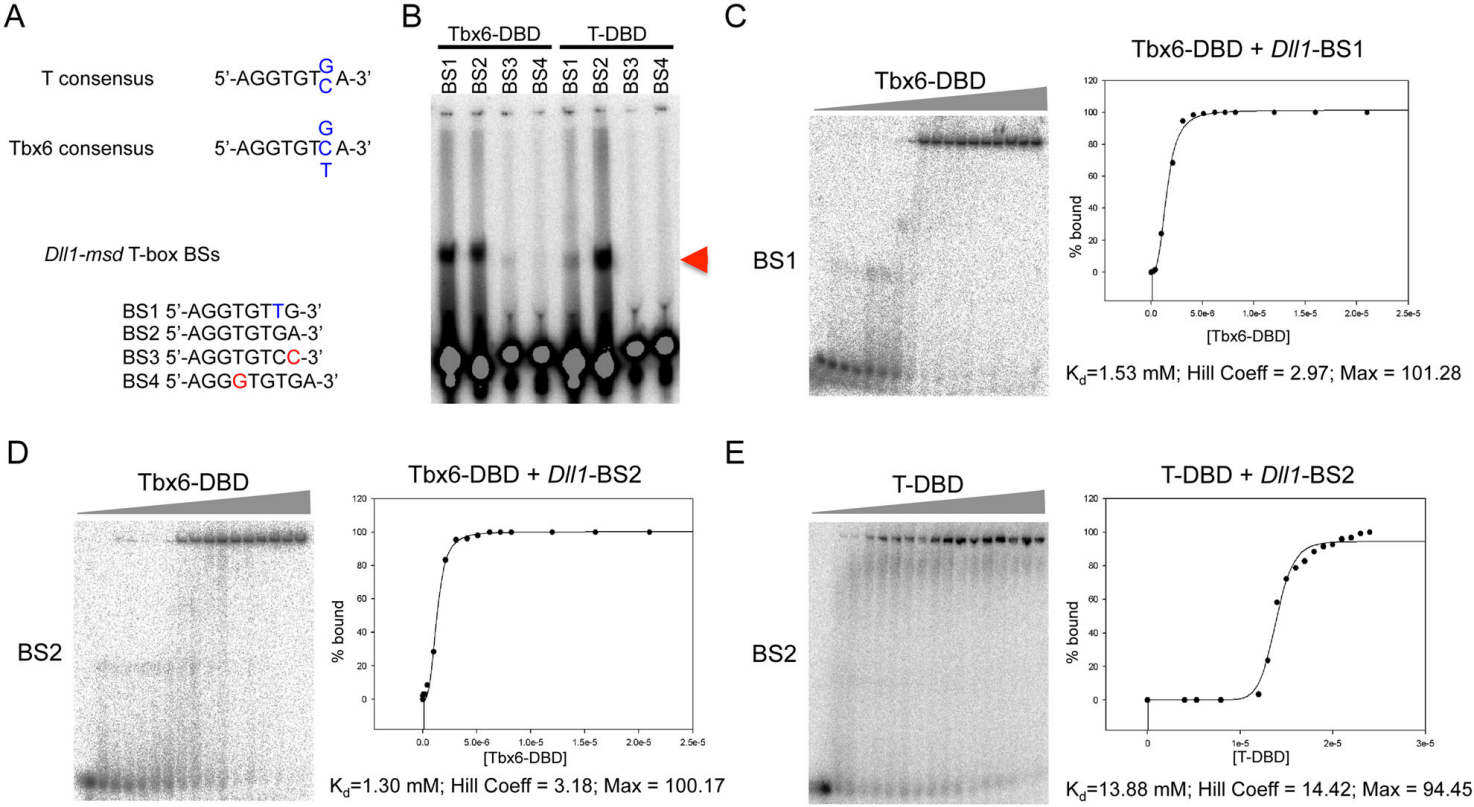
**Binding of Tbx6 and T DNA binding domains** **to the T-box binding sites in the *Dll1-msd* enhancer.** (A) EMSAs using the DBDs of Tbx6 and T and the four T-box binding sites (BS) found in the *Dll-msd* enhancer. The sequences for the T and Tbx6 consensus BS and the four BSs found in the *Dll1-msd* enhancer are shown with the variable seventh position in blue and mismatches in red. (B) Arrowhead indicates the shifted radiolabelled DNA. (C–E) Fuji BAS-2500 phosphoimages of quantitative EMSAs using increasing amount of His-Tbx6-DBD (range 0.21 nM–2.1 μM) or T-DBD (range: 4.0 μM–2.4 μM) added to a constant 10 pM of double-stranded labeled oligonucleotide corresponding to *Dll1-msd* BS1 or BS2. Percentage DNA bound versus concentration of protein was plotted and fitted to a three-parameter Hill equation to determine binding affinity (K_d_), Hill co-efficient, and maximum percentage bound (Max).

We previously showed using electrophoretic mobility shift assays (EMSAs) that full-length Tbx6 can bind both T-box binding site (BS) 1 (5′-AGGTGTTG-3′) and BS2 (5′-AGGTGTGA-3′) in the *Dll1-msd* enhancer (White and Chapman, 2005). Here we test the affinities of the DNA binding domains (DBD) of T and Tbx6 for the four putative T-box BSs in this enhancer (Fig. 1A). Similar to the full-length Tbx6 protein, the Tbx6-DBD could shift both BS1 and BS2 (Fig. 1B). The T-DBD could also shift both BS1 and BS2, however, shifting of BS1 appeared less effective (Fig. 1B). To determine the binding affinities of the T- and Tbx6-DBDs for BS1 and BS2 we used a quantitative EMSA approach whereby increasing amounts of the Tbx6-DBD or T-DBD were added to a constant, limiting amount of radiolabeled BS1-4 (Fig. 1C–E). Because the DNA concentrations were negligible compared to the protein, the protein concentration required to bind half the DNA was taken as an approximation of the disassociation constant, K_d_ (Harada et al., 1994). The K_d_’s of Tbx6-DBD for BS1 and BS2 were similar, at 1.53 µM and 1.30 µM, respectively. The T-DBD had a tenfold lower affinity for BS2, with a K_d_ of 13.88 µM. The binding affinity of T-DBD for BS1 could not be measured, as our protein preparation did not allow for high enough concentrations to achieve enough data points to fit to a curve. The Hill co-efficient of Tbx6-DBD was 2.97 and 3.18 at BS1 and BS2, respectively, and 14.42 for T-DBD at BS2. Strong cooperativity was observed for both Tbx6-DBD and T-DBD, as determined by a Hill co-efficient value greater than one. These results demonstrate that T has a lower affinity for the T-box BSs found in the *Dll1-msd* enhancer.

### T and Tbx6 transcriptional activities at synthetic and endogenous enhancers

**Fig. 2.**
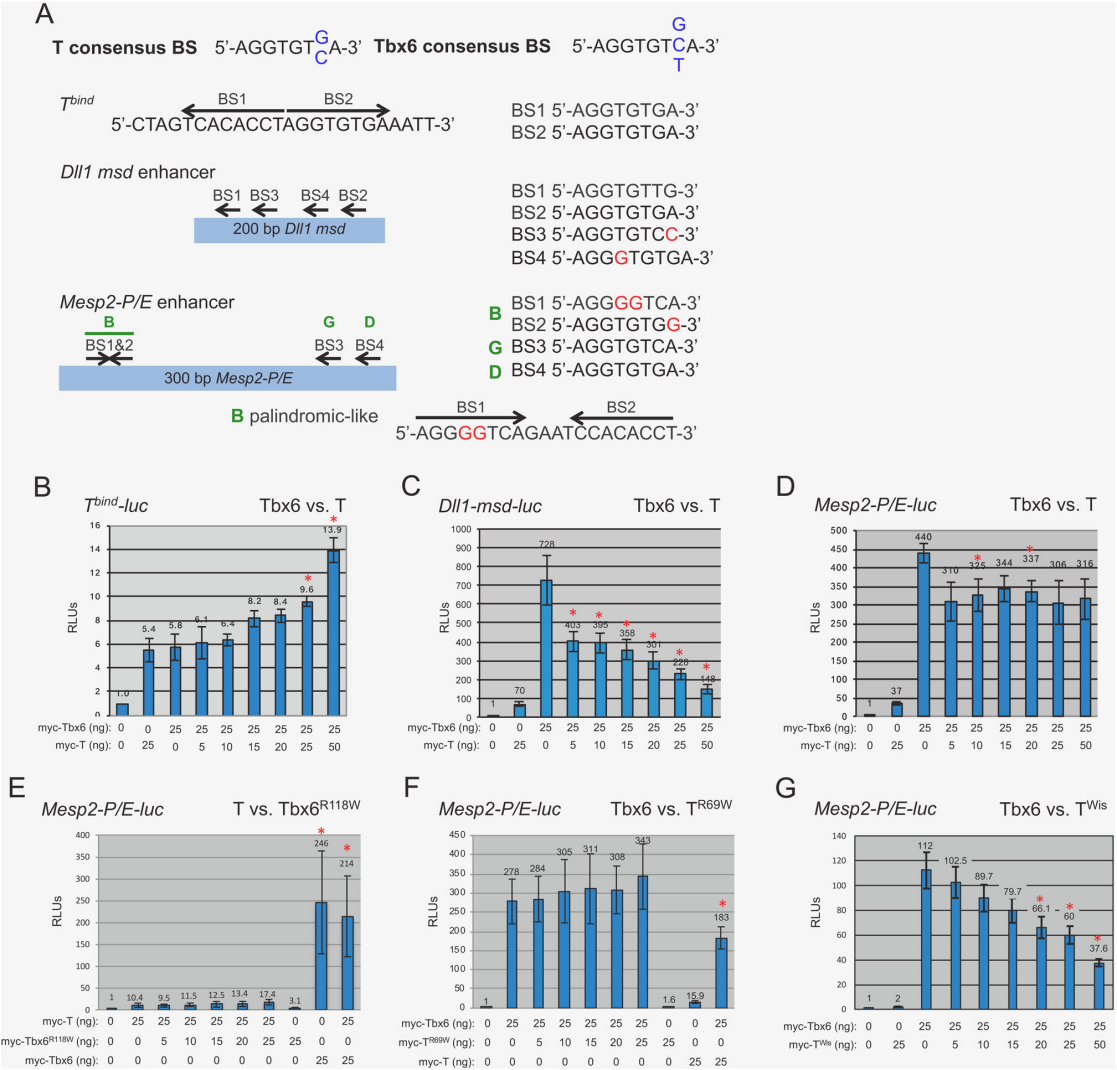
**Luciferase assays.** (A) Graphical representations of the enhancers used for luciferase assays with the T and Tbx6 consensus binding sites and the T-box binding sites found within the enhancers. Mismatches are denoted in red. (B–G) graphical analyses of relative luciferase units (RLUs) produced from transfecting the specified amount of the protein expression vector(s) with either the (B) T^bind^*-luc*, (C) *Dll1-msd-luc*, or (D–G) *Mesp2-P/E-luc* reporter vector. Empty protein expression vector served as a negative control and was set to 1. T^R69W^ and Tbx6^R118W^ are full-length proteins with a single amino acid change in the DBD. Competition luciferase assays were performed by adding increasing amounts of myc-T, myc-Tbx6, myc-T^R69W^, myc-Tbx6^R118W^, or myc-T^Wis^ to a constant amount of myc-Tbx6 or myc-T, as indicated. Red asterisks above bars indicate *P*<0.05.

Given these differences in the binding affinities of  T and Tbx6 for the sites with the *Dll1* enhancer, we next wanted to compare their transcriptional activities at several T-box enhancers, including the 24 bp palindromic T-bind site (T^bind^), a ∼200 bp region of the *Dll1-msd* enhancer (*Dll1-msd*), and a ∼300 bp promoter/enhancer region of *Mesp2* (*Mesp2-P/E*) each cloned upstream of a minimal promoter-luciferase (-luc). Both the *Dll1-msd* and *Mesp2-P/E* enhancers contain four putative T-box binding sites (Fig. 2) (White and Chapman, 2005; Yasuhiko et al., 2006, 2008). We generated N-terminal myc-tagged full-length T and Tbx6 expression constructs to characterize the activity of T and Tbx6 at these enhancers. *T^bind^-luc* co-transfected into HEK293T cells with equivalent amounts of myc-Tbx6 or myc-T plasmids revealed that both myc-T and myc-Tbx6 activate transcription weakly from this enhancer, approximately 5.8- and 7.5-fold over background, respectively (Table 1).

**Table 1.**
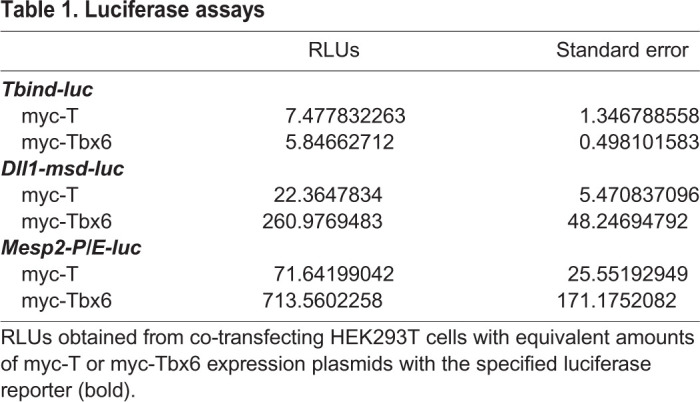
**Luciferase assays**

The four T-box binding sites in the *Dll1-msd* enhancer are clustered within a 100 bp region. As confirmed by our binding affinities results, BS1 and BS2 match the Tbx6 consensus site, while only one matches the T consensus (Fig. 2). *Mesp2* is a confirmed downstream target of Tbx6, and is expressed in the anterior portion of the PSM (Yasuhiko et al., 2006). *Mesp2-P/E* contains four putative T-box binding sites within 300 bp upstream of the start of transcription and the endogenous promoter sequences (Fig. 2). Two of these sites match both the T and Tbx6 consensus binding sites (sites D and G from Yasuhiko et al., 2008), while the other two are found in a palindromic-like configuration (site B) that contains mismatches compared to the T and Tbx6 consensus sites. At least two of the sites (B, D or G) are required for expression in transgenic embryos and for Tbx6 activation in luciferase assays (Yasuhiko et al., 2008). Contrary to the T-bind synthetic enhancer results, myc-T and myc-Tbx6 activated at different levels from the *Dll1-msd* and *Mesp2-P/E* endogenous enhancers, with myc-Tbx6 consistently activating tenfold higher than myc-T (Table 1 and Fig. 2).

For each of the luciferase reporters we further tested whether we could detect evidence of competition between T and Tbx6. For these experiments, we performed luciferase assays transfecting a constant amount of the myc-Tbx6 expression plasmid with increasing amounts of myc-T expression plasmid. Because the levels of activation from the *T^bind^-luc* were not statistically different when myc-T or myc-Tbx6 were added separately, we predicted that we would not see a change in relative luciferase units (RLUs) with increasing levels of myc-T. As predicted, the addition of increasing amounts of myc-T to a steady amount of myc-Tbx6 for the *T^bind^-luc* were not statistically different from Tbx6 alone until the highest levels of myc-T (25–50 ng) were added to a constant amount of myc-Tbx6 (25 ng). As previously described, we observed a tenfold difference in T versus Tbx6 transcriptional activity at the *Dll1-msd* and *Mesp2-P/E* enhancers when myc-T and myc-Tbx6 expression plasmids were used separately (Fig. 2C,D). We therefore hypothesized that if myc-T could compete with myc-Tbx6 at these enhancers then increasing the amount of myc-T relative to a constant amount of myc-Tbx6 would reduce the RLUs. In these experiments, we observed a statistically significant decrease in RLUs with increasing amounts of myc-T for *Dll1-msd-luc* (Fig. 2C). Less robust results were observed at *Mesp2-P/E*, for which statistically significant differences were only occasionally detected with the addition of myc-T, but this did not always correlate with the higher amounts of myc-T added (Fig. 2D). We next tested whether the DNA binding domain was necessary for competition by constructing myc-tagged full-length T and Tbx6 expression constructs that contain a single point mutation in the respective DBD, designated T^R69W^and Tbx6^R118W^. The point mutation changes a highly conserved arginine (polar amino acid) to a tryptophan (non-polar). This arginine makes polar interactions with DNA (Müller and Herrmann, 1997) and therefore a change from a charged to a non-polar amino acid is predicted to interfere with DNA binding. Mutations at the corresponding site in *Drosophila* T-box factor Omb fails to bind DNA (Sen et al., 2010). As predicted, the DBD mutants failed to activate or repress transcription in luciferase assays when used alone and did not compete when added with the converse wild-type T or Tbx6 (Fig. 2D,E). These results confirm that an intact and functional DBD, the T-domain, is required for competition between T-box factors in transcriptional assays.

### Upregulation of Tbx6 leads to T-like phenotypes

**Fig. 3.**
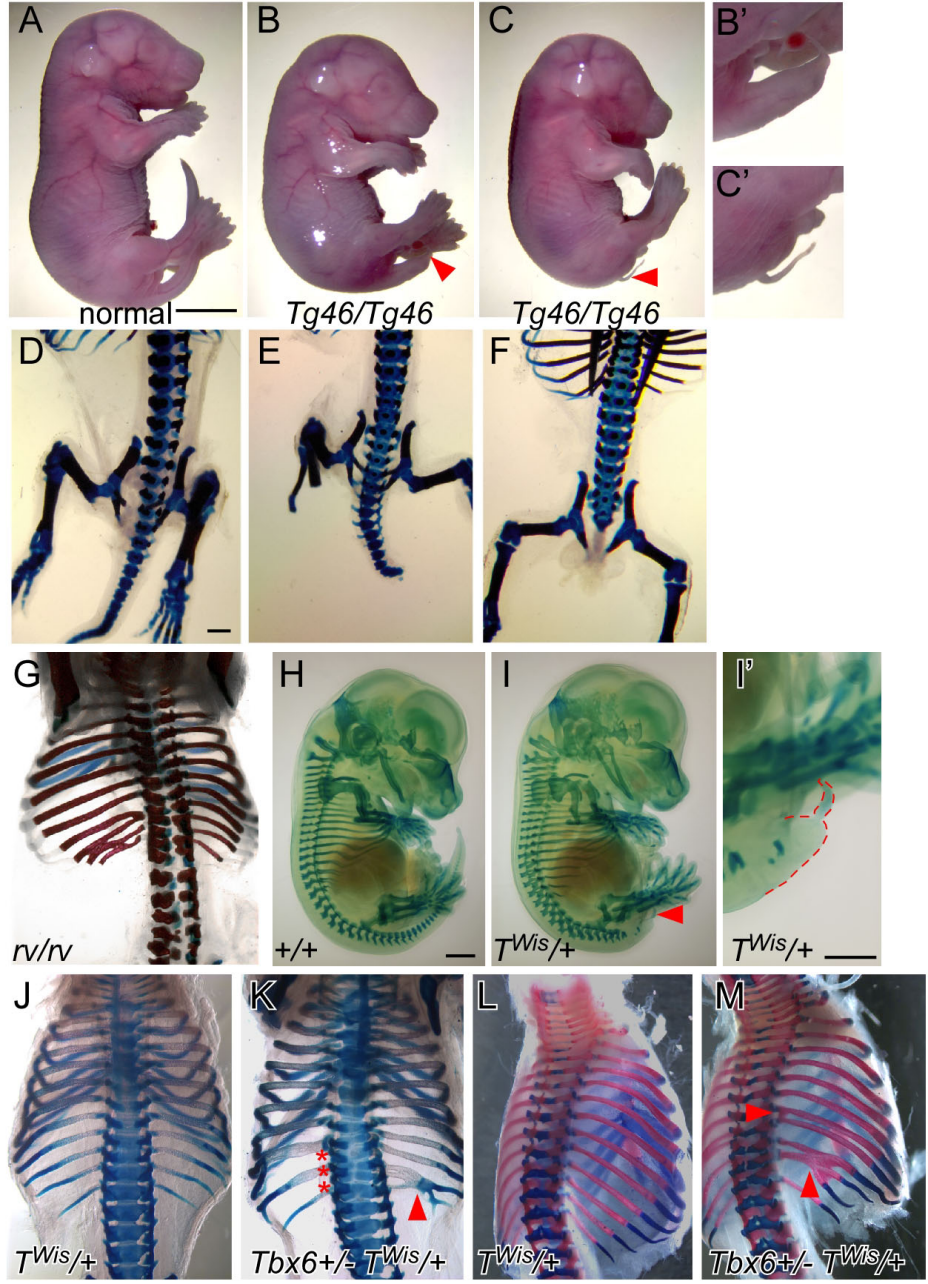
**Embryonic abnormalities resulting from** **altering Tbx6 and T activity.** (A–F) Gross morphology and skeletal preparations of embryos dissected at e18.5 from *Tg46/+* intercrosses. *Tg46/Tg46* embryos show truncated axes, ending either in a short (B,B′) or filamentous tail (C,C′), and are smaller than their normal littermate. (D–F) Posterior regions of Alcian Blue/Alizarin Red stained skeletons highlight the axis truncation and loss of caudal vertebrae. (G) Thoracic and lumbar regions of Alcian Blue/Alizarin Red stained *Tbx6^rv/rv^* e18.5 embryo displaying abnormal vertebral and rib morphology, including fusions of the ribs. (H,I) Alcian Blue skeletal preparations of e13.5 embryos dissected from crosses of *+/+* and *T^Wis^/+* mice. The truncated axis of the *T^Wis^/+* resembles the Tbx6*^Tg46/Tg46^ (Tg46/Tg46)* embryos, however, these embryos are the same size as their wild-type (*+/+*) littermate. The truncated tail of the *T^Wis^/+* embryo is marked by a red arrowhead (I) and shown in higher magnification and outline in red in panel I′. Alcian Blue/Alizarin Red staining of e16.5 (J,K) and e17.5 (K,L) embryos dissected from *T^Wis^/+ x Tbx6+/−* crosses. Note the fusions of vertebrae (red asterisks) and ribs (red arrowheads) in the *T^Wis^/+ Tbx6+/−* skeletons but not in the *T^Wis^/+* skeletons. Magnification bars represent 3 mm (A–C), 600 μm (D–G,J–M), 1 mm (H,I), 1 mm (I').

In mice, homozygous loss of *Tbx6* results in the mis-patterning of anterior somites, the formation of ectopic neural tubes at the expense of posterior somites, an enlarged tailbud and embryonic lethality by e12.5 (Chapman and Papaioannou, 1998). Approximately half of *Tbx6* heterozygous embryos display defects in the formation of the atlas and axis, while a quarter have defects in 1–2 sacral vertebrae (Sparrow et al., 2013). The *Tbx6* hypomorphic mutation, *rib-vertebrae (Tbx6^rv^)*, is a mutation in the regulatory region of Tbx6 resulting in less than heterozygous levels of *Tbx6* expression in *Tbx6^rv/rv^* embryos and mice with fusions of ribs and vertebrae and a shortened axis (Watabe-Rudolph et al., 2002; White et al., 2003). To further explore the phenotypic consequences of altering *Tbx6* expression levels, we utilized our *Tbx6* transgenic line, *Tbx6^Tg46^*, that harbors a transgene containing the entire *Tbx6* coding region along with upstream and downstream sequences required for proper temporal and spatial expression of *Tbx6* (White et al., 2005, 2003). The *Tg46* transgene expresses *Tbx6* RNA at lower than heterozygous levels and thus fails to rescue the *Tbx6* mutant phenotype; *Tbx6-/- Tbx6^Tg46/+^* embryos display fusions of vertebrae and ribs similar to the *Tbx6* hypomorph, *Tbx6^rv^*^/*rv*^ (White et al., 2003). Embryos hemizygous for the *Tg46* transgene (*Tbx6^Tg46/+^)* on a wild-type background are phenotypically normal, except for an occasional (∼5%) kinked tail. We tested the consequence of increasing the level of Tbx6 by homozygosing the *Tg46* transgene. Interestingly, *Tbx6^Tg46/Tg46^* embryos have severely truncated axes that terminate in a filamentous tail structure, with malformed or absent vertebrae in the filamentous tail regions (Fig. 3B,C). The small tail phenotype is noticeable by e9.5–e10.5, with tailbuds expressing lower levels of *T* suggesting a loss of progenitor cells necessary for caudal extension (Fig. S1D). By e15.5, these *Tbx6^Tg46/Tg46^* embryos are noticeably smaller than wild type and their hemizygous littermates with a high proportion dying perinatally for unknown reasons as all of the organs appear normal (Fig. 3A–C). In addition to the previously published consequences of under-expressing Tbx6 (Watabe-Rudolph et al., 2002; White et al., 2003), the above results show that there are also phenotypic consequences for over-expressing *Tbx6*.

### Tbx6 protein levels vary in different genetic backgrounds

Western blot analysis was used to quantitate Tbx6 protein levels in e10.5 tailbuds from *Tbx6^rv^*^/*rv*^, *Tbx6+/−*, and *Tbx6^Tg46/Tg46^* embryos, in addition to their respective wild-type background strains (Fig. S1). Interestingly, Tbx6 levels varied among the different background strains: C57Bl6/J (*Tbx6^rv^*^/*rv*^ background) had the lowest levels of Tbx6 protein, followed by mixed C57Bl6/J/129SvEv (*Tbx6+/−* background) and finally FVB/N (*Tbx6^Tg46/Tg46^* background) had the most. Tbx6 protein levels were also variable among the different genotypes: *Tbx6^rv^*^/*rv*^ tailbuds expressed the lowest, followed by a slight increase in *Tbx6+/−* tailbuds, and the greatest amount in *Tbx6^Tg46/Tg46^* tailbuds (Fig. S1). One caveat of this experiment is the low actin levels to which the *Tbx6^Tg46/Tg46^* Tbx6 protein levels were normalized, which could skew the Tbx6 protein levels such that they appear significantly higher than they actually are. We next used immunocytochemistry to confirm western blot results (Fig. S1). While Tbx6 protein appeared to be properly localized in the tailbuds of embryos across the various genetic strains, different staining intensities were observed that are consistent with the varying levels detected by western blotting. The tailbud sizes were also variable across the different genotypes. The decreased level of Tbx6 in *Tbx6^rv^*^/*rv*^ embryos results in an enlarged tailbud, while homozygosity for the *Tg46* transgene results in a smaller tailbud compared to their wild-type controls (Fig. S1C,D). Tbx6 was found throughout the enlarged *Tbx6^rv/rv^* tailbud apart from the ectopic neural tissue, which did not express Tbx6 (Fig. S1C). Tbx6 protein appeared throughout the reduced tailbud of the *Tbx6^Tg46/Tg46^* embryos, suggesting that even though there are fewer cells in the *Tbx6^Tg46/Tg46^* tailbud they express higher levels of Tbx6 compared to wild-type tailbuds. Altogether, these and previously published studies show that alterations in Tbx6 expression (less than heterozygous and greater than wild-type levels) can greatly affect the formation of the tailbud and somites; *Tbx6^rv/rv^* embryos express less than heterozygous levels of Tbx6 and consequently have fusions of ribs and vertebrae (Watabe-Rudolph et al., 2002; White et al., 2003), while *Tbx6^Tg46/Tg46^* express greater than wild-type levels, which affects tailbud size, axis extension and subsequent generation of somites and their derivatives.

### Dominant T allele, T^Wis^, interferes with Tbx6

The *Tbx6^Tg46/Tg46^* tails are phenotypically similar to those of embryos heterozygous for the *T* null or *T^Wis^* allele, i.e. ending in a filamentous tail stub (Fig. 2I). The *T^Wis^* mutation truncates the T protein in a regulatory domain required for its activity but leaves the DBD intact, thus *T^Wis^* is believed to be a neomorph, generating phenotypes more severe than the null allele; *T^Wis^/T^Wis^* embryos produce no somites, while the *T/T* embryos can generate up to nine anterior somites (Conlon et al., 1995; Herrmann and Kispert, 1994; Herrmann et al., 1990; Kispert and Herrmann, 1994; Shedlovsky et al., 1988). Like the *T* null, the *T^Wis^* mutation is epistatic to *Tbx6* (Chapman et al., 2003). The more severe *T^Wis^/T^Wis^* phenotype compared to that of the *T/T* (null allele) led to the hypothesis that the T^Wis^ protein blocks a related protein from binding the same DNA site(s) thus affecting transcription of target genes (Conlon et al., 1995; Herrmann and Kispert, 1994; Kispert and Herrmann, 1994). *Eomesodermin* and *Tbx6* are both co-expressed with T and are therefore candidates for this related protein (Arnold et al., 2008; Chapman et al., 1996; Russ et al., 2000). Here we genetically test whether T^Wis^ is interfering with Tbx6 function, by generating *Tbx6 T^Wis^* double heterozygous embryos (*Tbx6+/− T^Wis^/+*), thus genetically reducing the amount of Tbx6 (*Tbx6±+/−*) while expressing the T^Wis^ interfering protein (*T*^Wis^*/*+). To examine the phenotypes, we performed stains using Alcian Blue (cartilage) with or without Alizarin Red (ossified bone) of e15.5–17.5 skeletons. Indeed, eight out of fifteen *Tbx6+/− T^Wis^/+* embryos displayed fusions of several ribs and malformed vertebrae (Fig. 3K,M), resembling the *Tbx6* hypomorph, *Tbx6^rv/rv^* (shown in Fig. 3G), while only one severely affected *T^Wis^/+* embryo (*n*=10) displayed rib fusions (Table 2). If rib fusions and vertebral abnormalities in *T^Wis^/+ Tbx6+/−* embryos are simply due to a loss of T protein function and not to a T^Wis^ blocking function, then embryos heterozygous for *Tbx6* in combination with the *T* null allele should have the same phenotype as the *Tbx6+/− T^Wis^/+* embryos. Instead, we found no rib fusions or vertebral malformations in *Tbx6+/− T/+* embryonic skeletons (*n*=7; Table 2). This phenotypic effect of *T^Wis^* was specific to *Tbx6* as neither *wnt3a* nor *Dll1*, two genes functioning in this pathway (Dunty et al., 2008), had this effect when combined with *T^Wis^* (Table 2). These results suggest that T^Wis^ specifically blocks Tbx6 function. Using luciferase assays we further show that while T^Wis^ itself has no activation or repressive activity at *Mesp2-P/E-luc*, it can decrease the RLUs when increasing amounts of T^Wis^ expression plasmids are co-transfected with a constant level of Tbx6 expression plasmid (Fig. 2G).

**Table 2.**
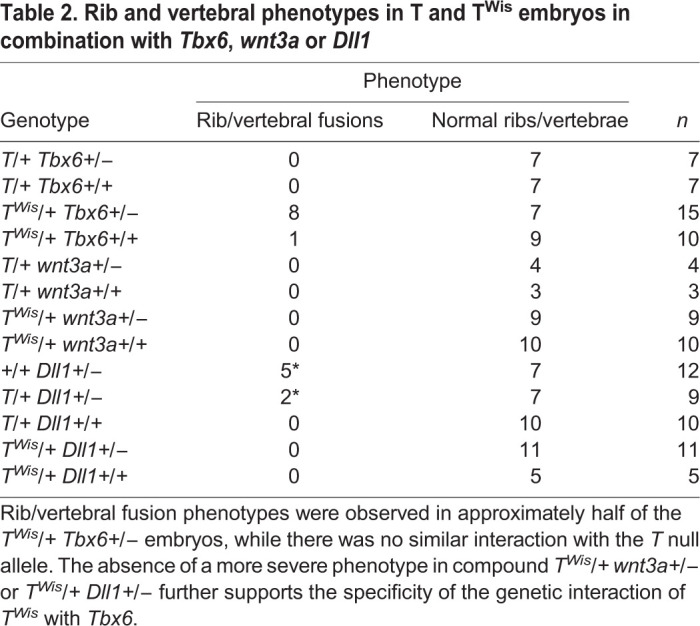
**Rib and vertebral phenotypes in T and T^Wis^ embryos in combination with *Tbx6*, *wnt3a* or *Dll1***

### Tbx6 cannot functionally replace T

**Fig. 4.**
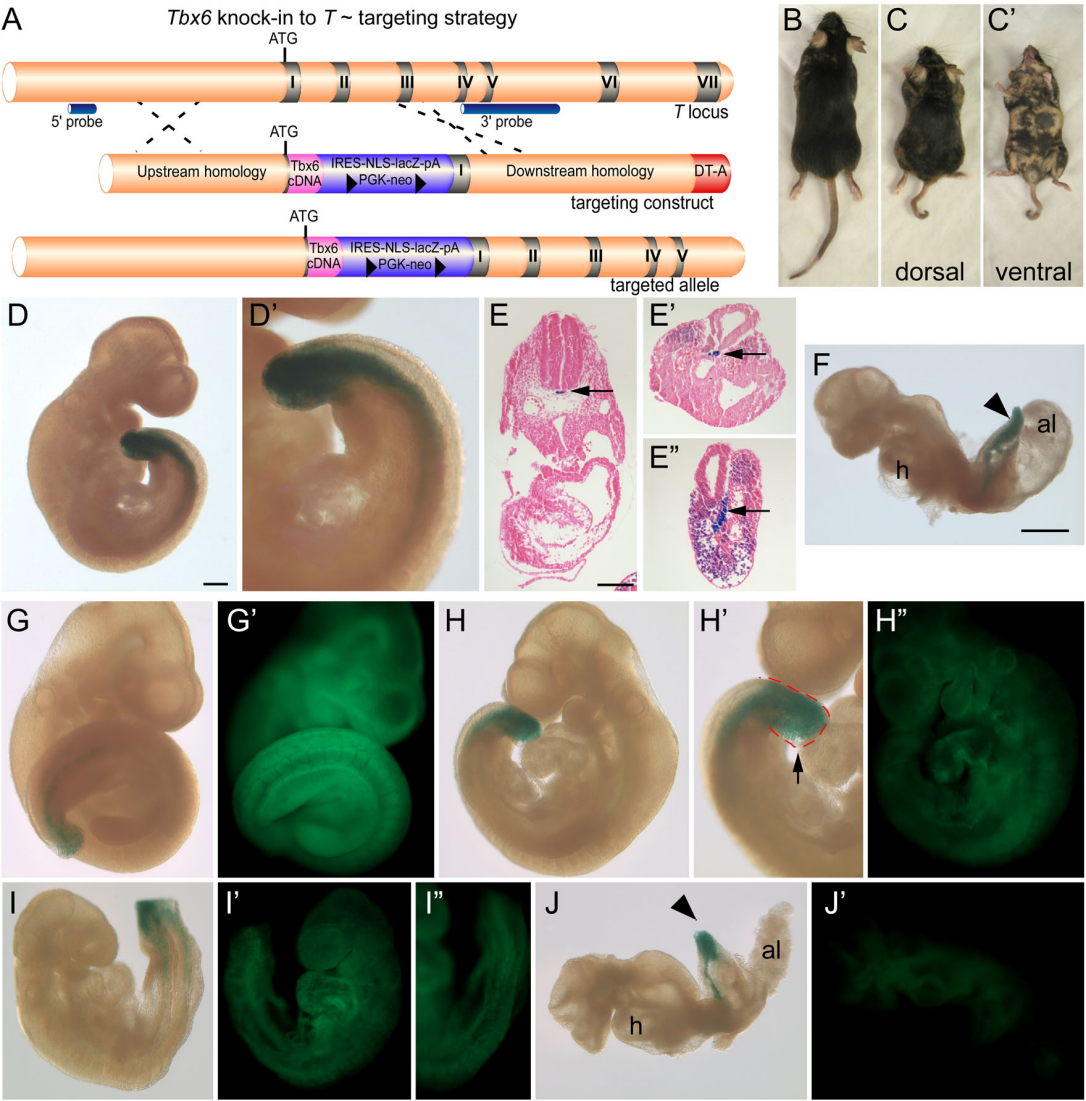
***Tbx6*** **knockin** ***T* targeting strategy and chimera** **phenotypes.** (A) Schematic of the targeting strategy to knock the *Tbx6* cDNA into the *T* locus at the initiating methionine *T^Tbx6ki^*. The positions of the IRES-lacZ-PGK-neo positive selection cassette, the diphtheria toxin A (DT-A) negative selection cassette, and 5′ and 3′ external probes for genotyping are indicated. (B,C) Dorsal views of littermate chimeric mice derived from injecting *T^Tbx6ki/+^* ES cells into C57 blastocysts. Mice in panels B and C represent ∼10% and 40% chimerism, respectively. (C′) Ventral view of chimera shown in panel C. Note the short axis and kinky tail of the higher percentage chimera (panel C) compared to its littermate (panel B). (D,D′) β-galactosidase staining of a chimeric embryo and representative sections (E–E″) showing the presence of the *lacZ* reporter activity in the PS and notochord (arrow), indicative of the *T* expression domains. (G–J) Chimeric embryos resulting from *T^Tbx6ki/+^* ES cell injections into GFP-expressing blastocysts were stained for β-galactosidase activity and imaged in bright field and GFP fluorescence (G′–J′). Panels G to J represent low (panel G,G′) to high percentage (panel J,J′) contribution of the *T^Tbx6ki/+^* ES cells in chimeric embryos as shown by reduced GFP from panels G′ to J′. Developmental defects include abnormal tail and somite morphology (H,I) and shortened axis and failure to turn (J) in the higher percentage chimeras. Magnification bars represent 200 μm (D,G–I), 150 μm (E), 700 μm (F,J).

While T and Tbx6 share similarities within the DBD and can bind similar sequences *in vitro* our current results show that they have different affinities for these binding sites, which may account for their differential activity in luciferase assays. Nevertheless, these factors can compete *in vitro* with competition being dependent on their T-domain, suggesting that they could have some redundant functions. Data from our lab along with others indicate at least some non-redundant functions. In *T/T* embryos, *Tbx6* is initially expressed, however, a mutant phenotype is evident by the time *Tbx6* expression is lost (Chapman et al., 1996). In *Tbx6* mutants, *T* expression is maintained in the bulbous tailbud, but this tissue does not form PAM (Chapman and Papaioannou, 1998). Thus, neither Tbx6 nor T appears to compensate for a loss of the other*.* However, this inability to compensate may simply be due to the level of T/Tbx6 protein expressed in mutant embryos. To further understand how similar or different T and Tbx6 function *in vivo* we undertook a knockin strategy in mice. The full-length *Tbx6* cDNA along with an IRES-nuclear localized *LacZ* and floxed *neo* selection cassette was knocked into the *T* locus at the initiating methionine (allele denoted *T^Tbx6ki^*, Fig. 4A). Two of the correctly targeted ES cells were injected into C57Bl6/J blastocysts and chimeric mice (*n*=30) were obtained. Interestingly, the chimeric mice obtained showed a low contribution from the ES cells as determined by coat color. One chimera with approximately 30–40% contribution from the ES cells had a short, kinky tail and shortened trunk compared to non-chimeric and low percentage chimeric littermates (Fig. 4C versus B). To determine whether high percentage chimeras were dying during embryogenesis we dissected chimeric embryos at e9.5. Chimeric embryos showed β-galactosidase activity in the notochord and tailbud in a T-specific manner, demonstrating that the knockin did not disrupt proper spatial expression from the *T* locus (Fig. 4D,E). However, abnormal phenotypes, including malformed somites and shortened axes, were observed in chimeric embryos (Fig. 4F). To correlate the ES cell contribution with the observed phenotypes, we injected *T^Tbx6ki/+^* ES cells into blastocysts ubiquitously expressing GFP and transferred the embryos to recipient females to allow for further development. Embryos were dissected at e9.5, stained for β-galactosidase activity and imaged embryos using both bright field and fluorescent microscopy. Increased ES cell contribution corresponded to lower GFP signal. Chimeric embryos with low to medium contribution had defects in tailbud morphology, which was often blunt and edemic, with higher ES cell contributions resulting in abnormally-shaped somites (Fig. 4G–I). Finally, embryos with the highest contribution were lethal at this stage; embryos failed to turn and had truncated axes (Fig. 4J). Therefore high contributions by *T^Tbx6ki/+^* ES cells that have only one functional copy of the *T* gene and express ectopic *Tbx6* in a *T*-specific manner lead to phenotypes indicative of disruption of PS function and somite formation. These embryonic defects presumably lead to the shortened axis and the kinked tail in the live born chimera (shown in Fig. 4C). These results suggest not only that Tbx6 cannot functionally replace T, but also that the levels of T and Tbx6 must be tightly regulated for proper mesoderm formation.

## DISCUSSION

T and Tbx6 are co-expressed in the primitive streak in addition to their unique areas of expression, *T* in the notochord and *Tbx6* in the presomitic mesoderm. Both T and Tbx6 are also thought to be transcriptional activators and to regulate at least one common target, *Dll1* (Hofmann et al., 2004; White and Chapman, 2005; Yasuhiko et al., 2006, 2008). Here we sought to understand how similar or different these related T-box transcription factors function *in vitro* and *in vivo*. Because T and Tbx6 can bind to very similar sequences and both can activate gene expression, we asked whether they are interchangeable if they are expressed in the correct places and times.

We tested the *in vitro* transcriptional activity of T and Tbx6 at a synthetic (*T^bind^*) enhancer. Approximately equal levels of activation by Tbx6 and T at the *T^bind^* enhancer was not surprising, as both T and Tbx6 have previously been shown to bind to this element with T binding as a dimer across the two half-sites and Tbx6 binding as two monomers to the two half-sites (Kispert and Herrmann, 1993; Müller and Herrmann, 1997; White and Chapman, 2005). Both T and Tbx6 regulate *Dll1* expression (Hofmann et al., 2004; White and Chapman, 2005). Here we show that while both T and Tbx6 can activate transcription from the *Dll1-msd* enhancer *in vitro*, Tbx6 serves as a better transcriptional activator. We further tested a second endogenous enhancer, *Mesp2-P/E*. Similar to results with the *Dll1-msd* enhancer, Tbx6 activated transcription at a tenfold higher level than T. As demonstrated by chromatin immunoprecipitation (ChIP), *Mesp2* is a confirmed Tbx6 target (Yasuhiko et al., 2006)*.* Although myc-T can activate the *Mesp2-P/E* enhancer *in vitro*, it is unlikely that it reflects a physiologically relevant event, since *T* is not expressed in the anterior PSM where *Mesp2* expression overlaps with Tbx6. However, it is possible that instead of activating, T may bind the *Mesp2* enhancer in the PS/tailbud and here serves to block Tbx6 from binding, thus repressing *Mesp2* transcription. This possibility would need to be verified by ChIP experiments. Differential activation of the *Dll1-msd* and *Mesp2-P/E* enhancers by T and Tbx6 can occur for several reasons that are not necessarily mutually exclusive. Tbx6 may simply be a stronger activator of transcription than T. Alternatively, T may require a co-factor(s) for more robust activity and this co-factor(s) may not be expressed in the HEK293T cells that were used for luciferase assays. Indeed, others have shown that both T and Tbx6 can synergize with the canonical Wnt signaling pathway to regulate *Dll1* expression (Hofmann et al., 2004). The tenfold difference in their activities at these endogenous enhancers, allowed us to test the hypothesis that T and Tbx6 can compete at target gene enhancers. In these experiments, increasing the amount of  T, while Tbx6 levels remained constant, resulted in a significant decrease in transcriptional activity, most noticeable using the common *Dll1* target. We showed that this competition requires a functional DNA binding domain by using expression constructs that coded for full-length T and Tbx6 proteins, but which had a single point mutation in the DBD that is predicted to interfere with DNA binding. While these studies support that T and Tbx6 are competing at the level of DNA binding sites, rather than competing for co-factors in these assays, it is possible that the point mutations interfere with binding to an unknown co-factor(s). The truncated T^Wis^ protein that retains a functional DBD but does not itself activate or repress transcription in these luciferase assays could still compete with Tbx6 further supporting our *in vivo* genetic data.

Although we did not test the importance of the individual T-box BSs in our luciferase assays, we did measure the binding affinities of T and Tbx6 at two sites within the *Dll1-msd* enhancer. We first found that both T and Tbx6 can shift two (BS1 and BS2) of the four BSs, but that T shifted BS1 less effectively than BS2. Quantitative EMSAs confirmed these findings and that Tbx6 bound to both BS1 and BS2 at approximately the same affinity, which for BS2 was tenfold higher than T's binding affinity. We could not measure the affinity of T for BS1 because it was too low at the concentrations of proteins used. These differences in T and Tbx6 affinities are consistent with the binding site preferences identified for T and Tbx6 using binding site selection assays (Kispert and Herrmann, 1993; White and Chapman, 2005). For example, *Dll1-msd* BS1 has a T in the seventh position just outside the core AGGTGT. Binding site selections revealed only a G or C at this position for T, while G, C, or T was preferred for Tbx6. Interestingly, our quantitative EMSAs revealed Tbx6 had a tenfold higher affinity than T at BS2, for which binding site selection experiments showed that both T and Tbx6 could bind the site. Preferences of T and Tbx6 for multiple BSs in specific arrangements, for example in a palindromic orientation as was originally identified for the T dimer (Müller and Herrmann, 1997), may contribute to the differences found in affinity for BS2. The identification of additional T and Tbx6 targets by ChIP would be needed to further explore this possibility. These differences in binding affinities of T and Tbx6 to the sites in the *Dll1-msd* enhancer may contribute to their differential transcriptional activities found *in vitro*.

Because T and Tbx6 can compete *in vitro*, we were curious whether this also occurs *in vivo* and used a variety of transgenic mice to explore this possibility. We observed axis truncation and tail dysmorphology phenotypes in the *Tbx6^Tg46/Tg46^* embryos, which express greater than wild-type levels of Tbx6 in its endogenous domains, i.e. the PS and presomitic mesoderm. This result suggested that increased levels of Tbx6 interfere with the function of another T-box protein, with T being a likely candidate as it is required for axis elongation and the similarities of *Tbx6^Tg46/Tg46^* and T heterozygotes. Alternatively, overexpression of Tbx6 may simply drive higher levels of a downstream target(s) and this then interferes with normal axis formation. Our genetic studies utilizing the *T^Wis^* allele revealed a genetic interaction with *Tbx6* in double heterozygous embryos, specifically fusions and malformations of ribs and vertebrae. Interestingly these same phenotypes were not observed in *Tbx6*; *T* null allele double heterozygotes, nor were similar phenotypes observed in embryos double heterozygous for *T^Wis^* and either *wnt3a* or *Dll1*, two other genes functioning in the PS to presomitic mesoderm pathway. These results suggest that the truncated T^Wis^ protein, which contains an intact DBD, can interfere specifically with Tbx6 function in the developing embryo. This is supported by our observation that *T^Wis^/+*, *Tbx6+/−* embryos share similarities with *Tbx6^rv/rv^* embryos (hypomorphic allele) that expresses lower than heterozygous levels of Tbx6. Together these results suggest that the T^Wis^ protein can specifically block Tbx6 function. Future RNA-seq experiments to examine changes in gene expression in these different genetic contexts could lead to a better understanding of the exact mechanism underlying these phenotypes. In addition, quantitative ChIP experiments to measure changes in T and Tbx6 occupancy at target genes in these different genetic contexts would show how co-expressed T-box proteins interact at the genome level. Nevertheless, our results indicate that over- or under-expression of Tbx6 leads to the abnormal formation of axial structures, specifically ribs and vertebrae.

Finally, we tested whether T and Tbx6 were functionally interchangeable by replacing T with Tbx6 in the developing mouse embryos. Despite the T-domains of these proteins sharing 53% identity and both being transcriptional activators, Tbx6 could not compensate for the single loss of T even in chimeras. In fact, high percentage chimeric embryos containing *T^Tbx6ki^* heterozygous cells share similarities with *T* null embryos, including truncated axes and malformed somites. This result indicates that T and Tbx6 behave differently, which could occur simply through differences in their preferences for binding sites in target genes, transcriptional activity, or a combination of the two. However, due to the severity of phenotypes in chimeric embryos using *T^Tbx6ki^* heterozygous cells, which resembled *T* null rather than *T* heterozygous phenotypes, we favor instead that there is some level of competition between the related factors. This competition is supported by our additional genetic studies that showed increasing Tbx6 levels using our *Tg46* transgene generates T-like phenotypes while genetic studies using the dominant *T* allele, T^Wis^, appears to compete with Tbx6. Altogether, these results suggest that controlling both the localization and the levels of these related transcription factors is critical for normal development.

## MATERIALS AND METHODS

### T6 and T DBD cloning and expression

The DBD region of T and Tbx6 [T: amino acids (aa) 41-224; Tbx6: aa 90-277] were PCR amplified and cloned into the PET151/D-TOPO (Invitrogen) producing a Histidine-tagged fusion protein. Transformed bacterial cultures were auto-induced, lysed and His-tagged fusion proteins were purified using nickel affinity purification followed by TEV protease digestion and a second round of nickel affinity purification to remove the Histidine tag. The protein was then further purified via anion exchange and size exclusion chromatography. Limited trypsin proteolysis revealed that >90% of isolated, purified proteins were correctly folded.

### EMSAs

Oligonucleotides were end-labeled with γ-^32^P-ATP using T4 polynucleotide kinase. Oligonucleotides were annealed and purified using Micro Bio-Spin P-30 Tris purification columns (Bio-Rad). The percentage of double-stranded versus single-stranded probe was determined with the percentage of double-stranded probe recovered being greater than 85% in all cases. Subsequently, the percentage of double-stranded probe for each experiment was standardized so equivalent amounts were used. All EMSA binding reactions were prepared in a final reaction volume of 10 µl in BBT buffer (25 mM HEPES pH 7.4, 75 mM NaCl, 1 mM DTT. 0.25 mM EDTA, 0.1% NP-40, 1 mM MgCl_2_, 10% glycerol, 10 µg/ml BSA). 0.1 mg/ml Poly dI-dC was added as a non-specific competitor. Binding reactions were incubated at room temperature for 20 min and loading on 4–6% non-denaturing PAGE (37.5:1) run in 1× TAE. Gels were dried unfixed, exposed to a phosphoimager screen and imaged on a Fuji BAS-2500 Phosphoimager. Oligonucleotides used: (bold letters indicate core T-box binding sequence) *T^bind^*: 5′-CTAGTC**ACACCTAGGTGT**GAAATT-3′*Dll1BS1*: 5′-TCACTGT**AGGTGT**TGCTGTCCTGT-3′*Dll1BS2*: 5′-TCCCG**AGGTGT**GATTCTTGGA-3′*Dll1BS3*: 5′GTGGATCC**AGGTGT**CCTCACTGGGCTGC-3′*Dll1BS4*: 5′-TGGATCCT**AGG**G**TGT**ACCTGACGGCTGC-3′

For quantitation, reactions were prepared as described above, except that increasing amounts of Tbx6-DBD (range: 2.1×10^−8–^2.1×10^−5^ M) or T-DBD (range: 4.0×10^−6–^2.4×10^−5^ M) were added to a constant, limiting amount of labeled *Dll1 BS1-4* oligonucleotides (10 pM) and incubated one hour at room temperature to ensure reactions were at equilibrium. Reactions were run on a 6% non-denaturing PAGE. Quantitation was performed as previously described (Harada et al., 1994). Briefly, the amount of free and bound DNA was quantitated using a Fuji BAS-2500 phosphoimager and analysis with ImageGauge software. Percentage of bound DNA was determined by the following formula: (Shifted DNA)/(Shifted DNA+Free DNA). The concentration of Tbx6-DBD or T-DBD was plotted versus the percentage of DNA bound. The data was fit to a three-parameter Hill equation using SigmaPlot software (equation: 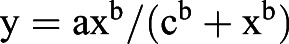, where a=the maximum value of y (percent bound), b=the Hill co-efficient, and c=K_d_).

### Plasmid constructs

Full-length *Tbx6* and *T* cDNAs were cloned in-frame with the N-terminal myc-epitope tag of the mammalian pCS expression vectors (Wehn and Chapman, 2010). To generate point mutations in the T and Tbx6 DBDs, we modified these pCS-myc-Tbx6 and -T expression vectors to change an arginine in the T-domain to a tryptophan (Tbx6^R118W^ and T^R69W^) using the QuikChange kit (Stratagene) following the manufacturer's instructions. To generate the truncated T^Wis^ protein, the region of the *T* cDNA encoding the first 345 amino acids was PCR amplified and cloned in-frame with the N-terminal myc-epitope tag of the pCS expression vector. The luciferase reporter vectors were all constructed in pGL4.10[luc] (Promega) except that a putative T-box binding site within the vector was changed (pGL4M*-β-globin-luciferase*). Enhancers included the 24 bp palindromic T-bind element, a ∼200 bp region of the *Dll1-msd* enhancer (*Dll1-msd*-luc) and a ∼300 bp region of the *Mesp2* promoter/enhancer (*Mesp2-P/E-luc*), which were cloned upstream of the β-globin minimal promoter-luciferase (Kispert and Herrmann, 1993; White and Chapman, 2005; Yasuhiko et al., 2006).

### Luciferase assays

HEK293T cells were chosen for luciferase assays because of their reliable transfection rate and their use for assaying transcriptional activity for multiple T-box proteins (Brown et al., 2005; Wehn and Chapman, 2010). 1×10^5^ HEK293T cells were plated per well in tissue culture-treated 96-well dishes, and transfected with Lipofectamine 2000 (Invitrogen) in suspension. 10 ng of the designated luciferase reporter plasmid was transfected per well along with 1 ng of *pRenilla Luciferase-CMV*, which served as an internal control. The amount of plasmid encoding myc-epitope tagged Tbx6, T, Tbx6^R118W^, T^R69W^ or T^Wis^ were as indicated, and empty pCS vector was added as necessary to maintain the same amounts of transfected DNA constant between samples. Twenty-four hours after transfection, cells were processed with Dual Glo luciferase reagent (Promega) according to the manufacturer's directions, and the intensity measured on a Berthold XS^3^ LB960 luminometer. Luciferase readings were normalized to the Renilla luciferase, and ratios were normalized to the luciferase plasmid transfected with an empty pCS3 expression vector control. Transfections were performed in triplicate in 96-well plates, and repeated at least once. Relative luciferase units (RLUs) and standard error were calculated over at least six data points. Statistical analyses were performed using one-way ANOVA tests.

### Mice

*Tbx6^tm1Pa^* (Chapman and Papaioannou, 1998), *Tg(Tbx6)46Dlc* (White et al., 2003), *T^Wis^* (Shedlovsky et al., 1988), *T* null (Kwan, Chapman, Behringer unpublished), *wnt3a^Tm1Amc^* (Takada et al., 1994) and *Dll1^Tm1Gos^* (Hrabĕ de Angelis et al., 1997) were utilized for genetic crosses. JAX stock #004353 mice *C57BL/6-Tg(UBC-GFP)30Scha/J* (Schaefer et al., 2001) were used to generate GFP-expressing blastocysts. Animals were mated and checked daily for the presence of a copulation plug. Noon on the day of the plug was considered e0.5 days post-coitum. Females were euthanized and embryos dissected from e9.5 to e18.5. All animal work was performed in accordance with the guidelines established by the University of Pittsburgh's Institutional Animal Care and Use Committee.

### Skeletal preparations

Skeletons from e14.5 to e18.5 embryos were stained with Alcian Blue with or without co-staining with Alizarin Red as described by Nagy et al. (2003), except that the staining was performed at 37°C.

### Western blotting

Embryonic tailbud tissue was dissected at e10.5 or HEK293T cells were transfected with the specified expression plasmids and the tissues/cells were homogenized in RIPA buffer. Bradford dye assays were performed to determine total protein concentration, and equal amounts of protein were loaded onto 7.5% SDS-PAGE gels, transferred to nitrocellulose, and blotted with rabbit anti-Tbx6 (1:2500) (White and Chapman, 2005), anti-9E10 (anti-myc, 1:500, Sigma-Aldrich) or anti-actin (1:1000, Cytoskeleton) in blocking buffer (TBTT containing 5% non-fat dry milk), and subsequently incubated in anti-mouse or rabbit HRP-conjugated secondary antibody (1:2500, Jackson ImmunoResearch), followed by ECL (Amersham) with Kodak Image Station quantification.

### Whole-mount immunocytochemistry

Immunocytochemistry was performed as described in Nagy et al. (2003). The Tbx6 N-terminal affinity purified antibody was used at a 1:500 dilution (White and Chapman, 2005). Goat anti-rabbit:HRP-conjugated secondary antibody (Jackson ImmunoResearch) was used at a 1:500 dilution and staining was performed in the presence of DAB, hydrogen peroxide and nickel chloride.

### Whole-mount *in situ* hybridization

Whole-mount *in situ* hybridization was performed as previously described by Wilkinson (1992) using antisense riboprobes for *T*. Hybridization and washes were performed at 63°C.

### Gene targeting

The Tbx6 knockin to *T* targeting construct was made by inserting the *Tbx6* cDNA at the start codon of the *T* gene, using 4.8 and 4 kb upstream and downstream homology regions from the *T* genomic region. An IRES-*lacZ*-floxed PGK-neo cassette was inserted after the *Tbx6* cDNA and a diphtheria toxin A cassette was inserted 3′ to the downstream homology for positive and negative selection, respectively. The linearized targeting construct was electroporated into R1 ES cells, selected and genotyped by Southern blot using 5′ and 3′ external probes according to standard techniques (Nagy et al., 2003).

### ES cell chimeras

Two of the targeted ES cell lines were injected into C57Bl6/J blastocysts or GFP-blastocysts, transferred to Swiss Webster pseudopregnant females, and allowed to develop *in vivo* either until birth (*n*=33 live born chimeras generated) or until e9.5 according to standard techniques (Nagy et al., 2003). Chimeric embryos (*n*=39) were dissected, fixed and stained for β-galactosidase activity (Ciruna et al., 1997) and either sectioned at 8–10 µm and co-stained with Eosin or imaged for GFP fluorescence.

## Supplementary Material

Supplementary information
