# Supplementary material for: Functionally distinct roles for T and Tbx6 during mouse development
Source: Biol Open. 2020 Aug 27;9(8):bio054692. doi: 10.1242/bio.054692 (PMC7473639; doi:10.1242/bio.054692)
Supplement: Supplementary information [file biolopen-9-054692-s1.pdf]

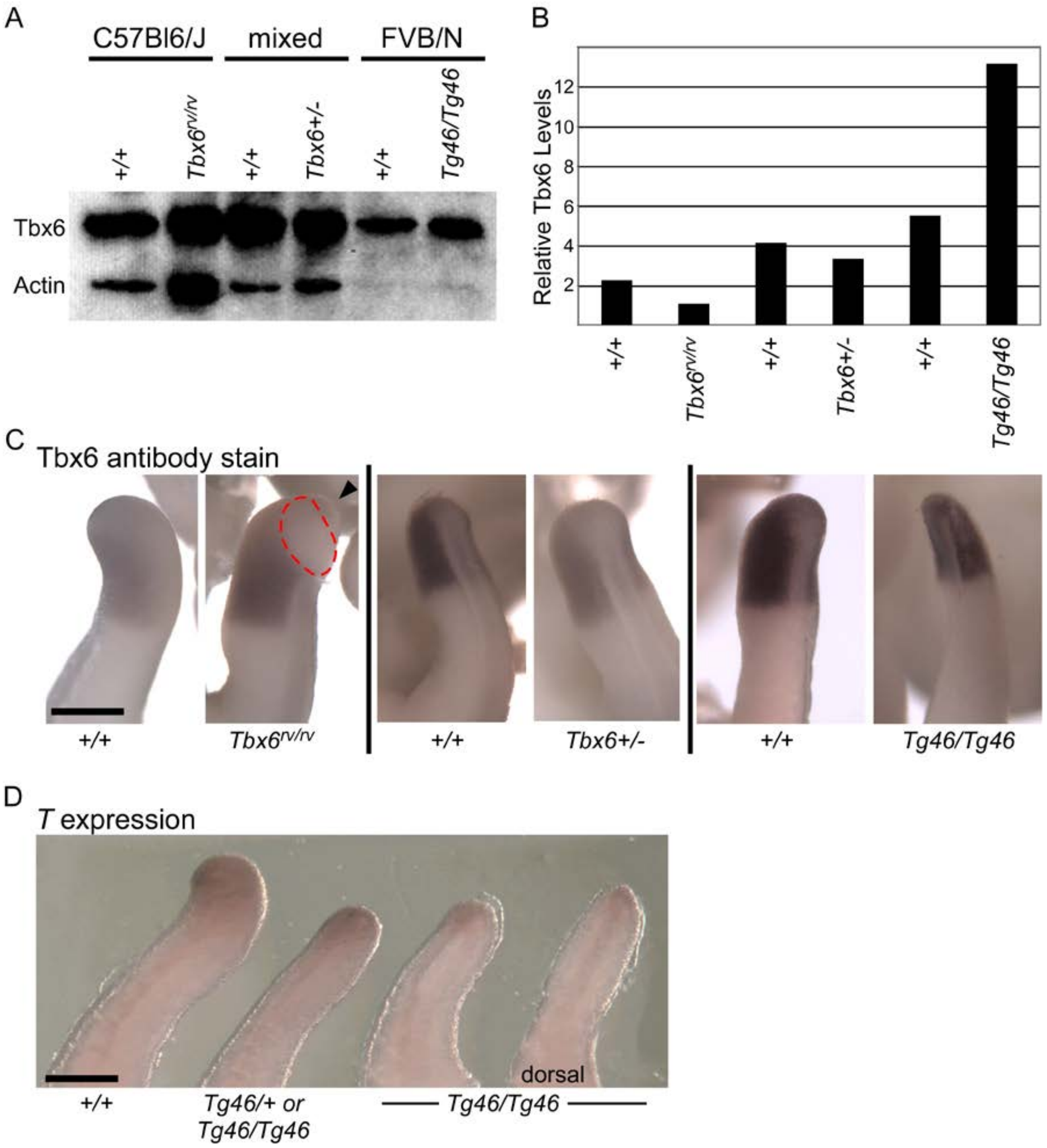

Figure S1. Quantitation and localization of Tbx6 across various genotypes and background strains. (**a**) Protein isolated from pooled e10.5 tailbuds from *Tbx6*$^{rv/rv}$, *Tbx6+/-*and Tbx6$^{Tg46/Tg46}$ *(Tg46/Tg46)* lines and their respective background strains as indicated was subjected to Western blot analysis using anti-Tbx6 and anti-actin antibodies. (**b**) Relative Tbx6 protein levels quantified using Kodak Image Station software and normalized to total actin levels. Increasing amounts of Tbx6 protein were found in the *Tbx6*$^{rv/rv}$, *Tbx6+/-*, *Tg46/Tg46* lines as well as their respective background strains. (**c**) Tailbuds from e10.5 embryos analyzed by immunohistochemistry for Tbx6 protein expression with the tailbud of each embryo shown. Tailbud morphology was

Biology Open • Supplementary information

affected across the various genotypes. Compared to their wild-type littermates, an enlarged tailbud was noted for the *Tbx6*$^{rv/rv}$ embryo, while a smaller tailbud was observed for the *Tg46/Tg46* embryo. Absence of Tbx6 staining within the *Tbx6*$^{rv/rv}$ tailbud (arrowhead and red dashed outline) is neural tissue. (**d**) Whole mount *in situ* hybridization of *T* expression in e10.5 embryos dissected from matings of *Tbx6*^{Tg46} heterozygotes (side and dorsal views). The small tail phenotype of *Tg46/Tg46* embryos is apparent by e10.5 as is the reduced expression of *T* compared to the wild type (+/+) control.
